# Proprioceptive art: How should it be defined, and why has it become so popular?

**DOI:** 10.1177/20416695221120522

**Published:** 2022-09-06

**Authors:** Charles Spence

**Affiliations:** Crossmodal Research Laboratory, Oxford University, Oxford, UK

**Keywords:** proprioception, bodily senses, art, installation, sensory deprivation

## Abstract

In recent years, there has been something of an explosion of interest in those artworks and installations that directly foreground the bodily senses. Often referred to as proprioceptive (or prop.) art, the question to be addressed in this narrative historical review is how it should be defined, and why has it become so popular? A contrast is drawn with examples of sculpture and/or tactile art. The entertainment/experiential element of such works cannot be denied, especially in an era where funding in the arts sector is so often linked to footfall. At the same time, however, a number of the works appear to be about little more than entertainment/amusement. One might wonder why such “edutainment” should be placed in the art gallery rather than, say, in a museum of science or illusion. Nevertheless, in the best cases, the foregrounding, or removal, of bodily sensations that proprioceptive artworks deliver can potentially help to connect people in an increasingly digital, online, mostly audiovisual, and hence in some sense disembodied contemporary existence. These issues are discussed in the context of the works of Carsten Höller, a prolific German installation and object artist.

## Introduction

There has been a rapid growth of interest in what has come to be known as “proprioceptive art” in recent decades. In this narrative historical review, I want to take a closer look at a number of those artworks that somehow directly stimulate the bodily senses, and consider various issues with the definition, conceptualization, and relative merit of proprioceptive art. I also want to address the question of why the interest in such works, many of which fall under the broad umbrella term of “relational aesthetics” (Bourriaud, 1997), has grown so much in recent years. I will assess the role of the experience economy, current funding models in the arts sector that promote a focus on popular entertainment, and also consider the possibility that the rise of proprioceptive art can be seen as an artistic response to the increasingly digital (and thus primarily audiovisual) and physically disconnected online existence that currently affects so many of us in society today. These issues are discussed in the context of the works of Carsten Höller, a prolific German installation and object artist, given the intriguing crossover that a number of his works present between art, science, and entertainment.

## Defining Terms

At the outset, it is important to note that there are various different ways of dividing up the so-called bodily senses (O’Shaughnessy, 1989; Paterson, 2021). For instance, Blakeslee and Blakeslee (2007, pp. 8–9) choose to discriminate between the categories of touch (including pressure and vibration),^1^ thermoception, nociception, proprioception (in which they incorporate kinesthesis—namely the sense of the body's motion; though see also Clark & Horch, 1986; Feldenkrais, 1990; Järvinen, 2006; Proske & Gandevia, 2012; Tuthill & Azim, 2018), and balance (relying on the vestibular system; see Gulden & Grüsser, 1998). The brain uses the information it receives from the proprioceptive receptors that are embedded within the muscles and tendons to measure stretch and thus to infer the location of the limbs while other receptors embedded in the cartilage between the skeletal joints help to keep track of load on, and the rate of slippage in, each of the joints. The latter signals are used to infer the speed and direction in which an individual's limbs are moving. Vestibular cues from the semicircular canals (which indicate rotational movements) and the otoliths (which indicate linear acceleration) provide an important source of information about the orientation of the body in space (i.e., motion, head position, and spatial orientation), and this sensory system is engaged by several of the works that will be discussed below. To this list, one might, of course, be tempted to add the many interoceptive senses that have been identified over the years (see Craig, 2002, 2009, for reviews).

As O’Shaughnessy (1995, p.175) has noted: “proprioception is attentively recessive in a high degree, it takes a back seat in consciousness almost all the time.” (cf. Armstrong, 1962; though see also Anscombe, 1962; Sherrington, 1906, pp. 335–343; Uytman, 1963). At the same time, however, it is also clear that proprioceptive signals contribute to the sense of body ownership (Walsh et al., 2011). What is more, there has also been growing interest in the notion of the role of bodily awareness in cognitive processes, a topic often referred to under the header of “embodied cognition” (Gallagher, 2005; Wilson, 2002). Indeed, many of the examples of proprioceptive art that will be discussed in this narrative historical review explicitly serve to foreground the bodily senses in the awareness of those who experience the works (cf. Paterson, 2021, for a historic overview of the scientific foregrounding of the bodily senses). Intriguingly, most require active, rather than passive, stimulation of the sense of touch (e.g., Nöe, 2006; Ratcliff, 2008).

One other potentially important distinction to be aware of when considering the role of the body in proprioceptive art is that between the notion of “body schema” and “body image” (e.g., Head & Holmes, 1911). However, although these terms have often appeared in the literature over the last century or so, their precise definition remains controversial. As Holmes and Spence (2005, p. 15) note: “The body schema and the body image are hypothetical constructs that are often used to describe or explain the results of a wide variety of experimental manipulations, neuropsychological disorders, and perceptual phenomena. Unfortunately, many different conceptualizations of “body schema” and “body image” are currently in circulation, and despite some valuable attempts to draw clear distinctions between these terms, confusion remains in the literature.” Hence, given the ongoing controversy and uncertainty in the cognitive neuroscience literature, these terms will not be used in this review.

One other point to bear in mind here is that the senses, which include the bodily senses, do not operate in isolation. Although it may not feel that way, perception is nearly always the result of the multisensory integration of various sensory cues, no matter whether they happen to be attended consciously or not (e.g., Bellan et al., 2017; Calvert et al., 2004; Schaefer et al., 2007; Taylor & McCloskey, 1991). As will become apparent later, the aesthetic pleasure of many of the proprioceptive artworks referred to in this review would seem to result from, and rely on, the multisensory integration of, and/or conflict between, multiple sensory cues (Bacci & Melcher, 2011; Sánchez Clemente, 2017; though see also Szubielska & Niestorowicz, 2020).

## Direct and Indirect Proprioceptive Artworks

In what follows, I will take an inclusive perspective on proprioceptive art. To be clear, proprioceptive art will be defined here as a label that can be applied to those works that include the direct first-person stimulation of any of the bodily (if not necessarily interoceptive) senses. At the same time, however, I will exclude any artworks that only stimulate the bodily senses indirectly, including the perception of dance amongst those who are dancers themselves (e.g., Bellan et al., 2017; Christensen et al., 2016; Franko & Lepecki, 2014; Montero, 2006, 2018; see also Christensen et al., 2018).^2^ This is not because of any doubt about their status as proprioceptive arts, but merely to keep the scope of the present manuscript within manageable limits.

Broadly speaking, the indirect route to stimulation of the bodily (interoceptive) senses perhaps ought to include the early Dutch still life with fruit paintings. The artist's intention in such cases was often to try and induce their viewers to salivate by rendering the glistening surface of the cut lemon as vividly as possible (see Leonhard, 2020).^3^ At the time, any artist who was able to elicit this response from their viewers would once have been considered to be at the top of their game. Relevant here, Berenson (1967, p. 40) has also written of how: “[The painter's] first business…is to rouse the tactile sense, for I must have the illusion of being able to touch a figure…before I shall take it for granted as real, and let it affect me lastingly” (see also Lopes, 2002). Indeed, Berenson argued for an “unconscious tactile ingredient in vision” and during genuine artistic experience imagined that bodily senses would somehow be aroused/engaged (Pallasmaa, 1996, 2011). Once again, though, all such indirect examples of interoceptive, or tactile, sensation in art will be excluded from this review of the field (e.g., Marks, 2002).

## On the Importance of Proprioceptive Intentions

**Figure 1.**
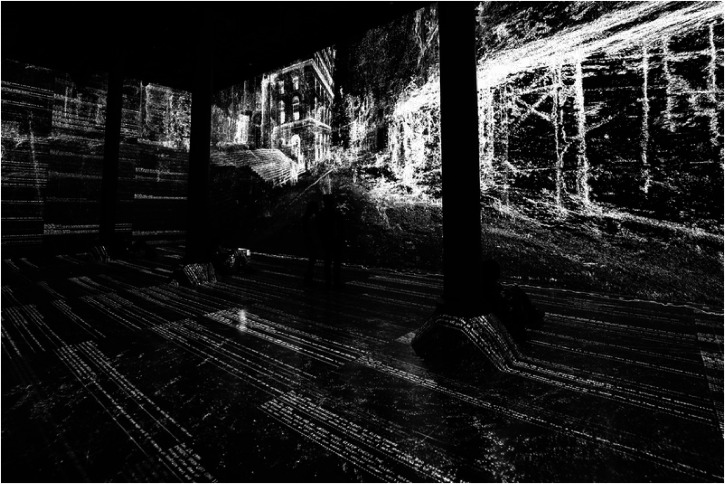
Artechouse NYC launches new must-see digital exhibition “Trust.” At certain points, when the immersive visual projections started scrolling, this led to proprioceptive awareness in the author. However, this would not count as an example of proprioceptive art because the proprioceptive element was not intentional, nor integral to the experience. [Reprinted from Madrigal (2022).].

According to the definition of proprioceptive art put forward here, the stimulation of the bodily senses should be somehow integral to the artist's work/intention. It is necessary to include this point in order to exclude those immersive artworks that stimulate the bodily senses, but where that stimulation would seem to have been incidental to the artist's overall aims and objectives. For instance, your author experienced one such example of indirect (and presumably unintentional) bodily awareness on a recent trip to the Art Fuse exhibit at Artechouse in New York (Madrigal, 2022). The work, which has now been presented in a number of cities around the globe, involved the immersive projection of a continuously changing array of images on the walls and floor together with an accompanying soundscape (see Figure 1). At one point in the show, when the angle of the projected visual horizon tilted, my attention was suddenly drawn to my own bodily sensations in order to determine whether my visual impression of the floor tilting was, in fact, correct (Fortunately, it was not.) In this case, my sense was that the sudden proprioceptive focus induced by the exhibit/installation was an incidental feature of the visual display, and not integral to the work's meaning. Hence, I do not think that this should be considered as an example of proprioceptive art.

**Figure 2.**
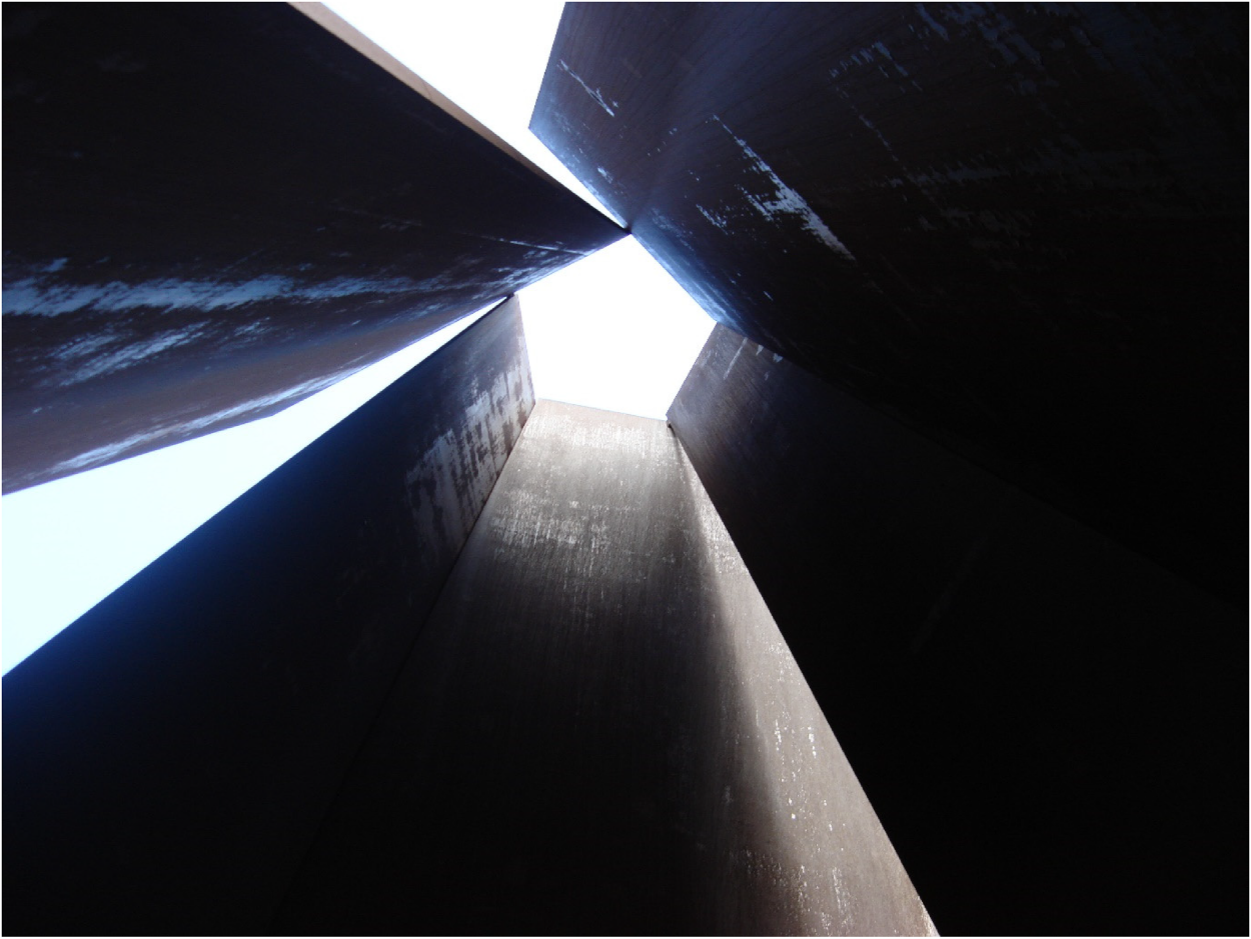
Richard Serra's Fulcrum, 1987. [Photograph: Oxyman, http://commons.wikimedia.org/.].

A somewhat more challenging body of work to decode is represented by Richard Serra's monumental sculptural works, such as *Fulcrum* (1987). According to Bacci (2011, p. 139), Serra's works “manage to almost enforce the public's touch by creating a bodily sensation of instability that requires haptic verification” (see Figure 2). At the same time, however, I fully recognize that this “intentionality” criterion represents a challenging border to police (see Adajian, 2008; Benovsky, 2020), given that intuiting the artist's intention can be difficult to ascertain, even in those cases where they are still alive (see also Daniels et al., 2010, on this theme). Furthermore, in many cases, the institutional perspective (cf. Blizek, 1974; Dickie, 1974) has, in recent decades at least, prohibited visitors from touching/interacting with the exhibits once they take on the status of the valuable art object (e.g., Chatterjee, 2008; Lupton, 2002).^4^ This approach was captured more than a century ago at Oxford's Picture Gallery in the Bodleian Library that read: “Touch what you like with the eyes, but do not see with the fingers” (Dickens, 1880, p. 153). Indeed, this was precisely what artist Miho Suganami reported (or complained about) happening to a number of her works such as “Have you touched your hands” (Suganami, 2003; see also Suganami, 2001).

## Tactile Art

Although the sculpture is typically considered a primarily visual art form nowadays, it is worth noting how some artists have intended for their works to be experienced through touch. Just take, for instance, Brancusi's sculpture for the blind from the opening decades of the 20th century (Bacci & Dent, 2008; Classen, 2012; Cranston, 2003; Gallace & Spence, 2014; cf. Johnson, 2002; Zuckert, 2009). In this case, the large marble egg was first displayed in a cloth sack (to prevent vision) and a couple of holes cut for the direct haptic exploration of the work. At around the same time, the Italian Futurists were also interested in educating the sense of touch through their artworks. According to Filippo Tommaso Marinetti, the founder of this artistic movement, “Tattilismo” (or the “Art of Touch”), was a multisensory evolution of Futurism (Marinetti, 1921a). Marinetti wanted to enhance the skin's sensitivity, which he described as “still a mediocre conductor of thought,” through the haptic experience of “tactile boards” (these were artworks made of different materials such as tinfoil, sponge, feathers, etc.), referred to as “hand journeys” (Marinetti, 1921a, 1921b). One famous example was Marinetti's *Sudan-Parigi* (“Sudan-Paris”; 1922, mixed materials, Geneva, private collection).

More recently, the North American artist, Rosalyn Driscoll (2020), has written of how she would like for her sculptures if not to be touched, at the very least to evoke imagined tactile sensations, in this case, elicited by the range of materials that are incorporated in her works. In particular, she writes that “It [tactile art] is intimate, drawing us into relationship with what we are touching. It is active rather than passive, requiring us to reach out and explore. It grounds the experience in perception rather than concept. Aesthetic touch deepens our knowledge of sensuous reality. We recognize an apple by looking at its colors, shape, and size; by touching it, we come to know its weight, mass, temperature, texture, and ripeness. If we are touching a sculpture, we feel the massing of forms, the texture and temperature of surfaces, the qualities of materials, and the nature of spaces.” (http://www.rosalyndriscoll.com/pages.php?which_page=book_introduction; see also Driscoll, 2011).

The above works would all seem to represent examples of what might be called tactile art. Importantly, the full appreciation of these works may well require the viewer's active bodily engagement. That said, in contrast to the works of proprioceptive art that will be discussed below, the focus in such cases is very much on the feel of the object itself, rather than on bodily sensations/experience. It may be said that the aesthetic pleasure in such works would appear to result either from the “aesthetic aha” (Muth & Carbon, 2013; Muth et al., 2019), as in the case of some of Miho Suginami's works, and/or, on occasion, from the pure pleasure of touching, or interaction with, the objects concerned (Jakesch et al., 2011; Soranzo et al., 2018).

## Proprioceptive Art

One of those whose work is perhaps most closely associated with the field of proprioceptive art is that of Belgian-born German installation and object artist Carsten Höller (Lindblad, 2012). A number of this former “mad scientist's”^5^ artworks deliberately engage proprioception, kinesthesis (Alexander, 2017; Russeth, 2011), and/or vestibular sensations in a seemingly playful manner.^6^ When considering the playful aspect of Höller's work, it is perhaps worth considering Conrads and Sperlich's (1963) statement in their book *Fantastic Architecture* that “What is called playful by those favorably inclined and is condemned as child's play by the serious…actually has profound significance. By detaching things from their familiar context, by considering them from hitherto unknown points of view, and by employing them without obvious purpose but out of sheer joy in totally different combinations, a resilient creative power is kept alive. And this is what mankind needs when the freezing point has been reached in a tradition which finds itself no longer capable of meeting a newly emergent problem.”

**Figure 3.**
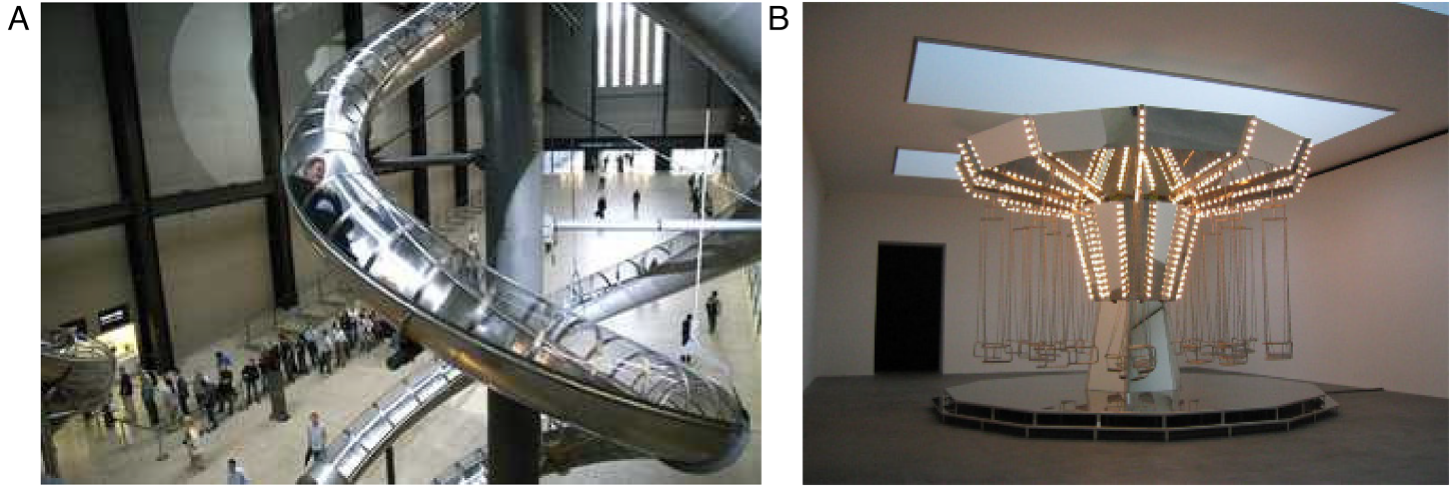
A) The Unilever Series: Carsten Höller: Test Site [Figure reprinted from https://www.tate.org.uk/whats-on/tate-modern/unilever-series/unilever-series-carsten-holler-test-site]; B) Mirror Carousel, also by Carsten Höller [Reprinted from https://www.wikiart.org/en/carsten-holler/carousel-mirror-2005].

Over the years, Höller has taken various fairground rides/concepts, including the helter-skelter and merry-go-round (colloquially known as the gallopers; e.g., Starsmore, 1975) and situated them in the context of the art gallery. Höller's various Slides exhibits have appeared in galleries around the world over the last decade or two), including famously at the Tate Modern's Turbine Hall in 2006/2007 (https://www.tate.org.uk/whats-on/tate-modern/unilever-series/unilever-series-carsten-holler-test-site) (see Figure 3). Meanwhile, Höller's “Mirror carousel” (2005) consists of a very slowly moving carousel that visitors were once again invited to sit on and enjoy (I will return to the importance of the speed, or lack thereof, a little later on). According to Schwartzman (2011; p. 32): “Höller engineers his work to penetrate through space and into the spectator's brain causing shifts in perception or heightened awareness of the act of perceiving.”

However, before taking a closer look at some of Höller's works, it should be stressed that this artist is by no means unique in attempting to foreground the experience of the bodily senses (see Table 1). While Ernesto Neto is listed in the table of examples of proprioceptive art, it is a little unclear whether many of the Brazilian artist's works should his work be classified as tactile, proprioceptive, or perhaps multisensory, given that scent is so often part of the total experience. Schwartzman (2011) documents a number of other artists and their works/installations that engage some configuration of the bodily senses. That said, Höller has perhaps done more than any other artist in the realm of proprioceptive art, and it is to his Experience exhibition that we shall turn next.

**Table 1.** Summary of Various Proprioceptive Artworks (and Associated Links).

“Mobile Feelings” (2002–2003) by Christa Sommerer & Laurent Mignonneau
(Sommerer & Mignonneau, 2002-2003)
“Embodied Sensations” (see Williams et al., 2021)
trg. Transient Reality Generators (2005). KIBLA Multimedia Center, Slovenia
(Schwartzman, 2011, p. 97)
Tomás Saraceno's “In Orbit” at K21, in Düsseldorf (Saraceno, 2015)
(https://www.kunstsammlung.de/en/exhibitions/tomas-saraceno-in-orbit-en;
https://www.tanyabonakdargallery.com/exhibitions/394-tomas-saraceno-in-orbit-
kunstsammlung-nordrhein-westfalen-k21-standehaus-dusseldorf/)
“White Bouncy Castle,” an installation from Dana Caspersen,
William Forsythe, & Joel Ryan (https://www.trendhunter.com/trends/white-bouncy-castle)
“Think Outside the Box” by Till Bödeker (2020; https://tillboedeker.art/; Lohe, 2020)
Various of the works of Ernesto Neto (see Schwartzman, 2011, pp. 84–85)

## The Experience Exhibition

Many of Höller's works were brought together for his Experience exhibition held in New York (New Museum to Present First New York Survey of Works by Carsten Höller, 2011), to mixed critical reviews. The exhibition included everything from “Upside Down Mushroom Room” sometimes referred to as “Giant Triple Mushrooms” (2000; see Schwartzman, 2011; pp. 32–33; Höller, 2009/2011), “Giant Psycho Tank” (1999), “Mirror Carousel” (2005), “Love Drug (PEA)” (1993/2011), “Untitled (Slide),” a 102-foot long slide that slices through three floors of the New Museum and a number of other works. Lindblad (2012) writes that “If we take the critics” evaluations as the guiding factor of whether or not Experience is a successful exhibition in a phenomenologically reflective sense, we can be sure it is not.”

According to the critic, Pollack (2011): “If the traditional work of art addresses the viewer as a thinking, aesthetically critical being, much of relational aesthetics (Bourriaud, 1997), including this show, addresses the spectator in a more familiar mode: that of the consumer.[…] Experience turns the museum into a fun-house, at a cost. What we lose is the critical faculty, which, in a way, brings us full circle: “Mr. Höller's is an exceptionally fun exhibition to visit, and a particularly difficult one to review.”^7^ Lindblad (2012) writes: “I would argue for a second look. After the effects of the exhibition—nausea, headaches, bruises, salty ears, shaky legs, exhilaration, or otherwise—have worn off, a focused reflection on the works is possible. The subjective, individual experience is, in the end, up to the viewer—the rat in the laboratory, the scientist in the lab coat, or the visitor in the carnival.”

One of the points to note here concerns the fundamentally multisensory nature of the experience of such fairground rides/attractions. Indeed, it is interesting to note how many writers have drawn attention to the sensory overload that is typically associated with the fairground/theme park phantasmagoria (e.g., see Addison, 1953; Lynn, 2006; Spence, 2022b, 2022c). This is undoubtedly an important part of the total entertainment experience that it is simply not possible to capture within the austere and respectful confines of the gallery setting. That said, it might even be wondered whether this may actually have been a deliberate strategy by the artist concerned to eliminate the extraneous sensory information in order to allow the visitor to focus primarily on their proprioceptive sensations. However, I am unaware of anyone having explicitly discussed this (including nothing from Höller himself).

According to another art critic: “Mr. Höller's [work] has indulged interactivity to an almost comical degree and disoriented viewers with crafty inventions and bizarre interventions in museum practice.” (Russeth, 2011). Russeth continues: “Mr. Höller has produced technically advanced works that ground users in their own private experiences, cutting them off from easy association with others. He has designed a pill to simulate love and glasses that flip the world upside down. At the New Museum's show, there will be one of the artist's trademarks Psycho Tanks, a sensory deprivation pool that renders participants weightless, and a mirrored carousel that provides surreal, fractured fun-house rides.”

## From the Theme Park, Fairground, and Science Lab to the Art Gallery

**Figure 4.**
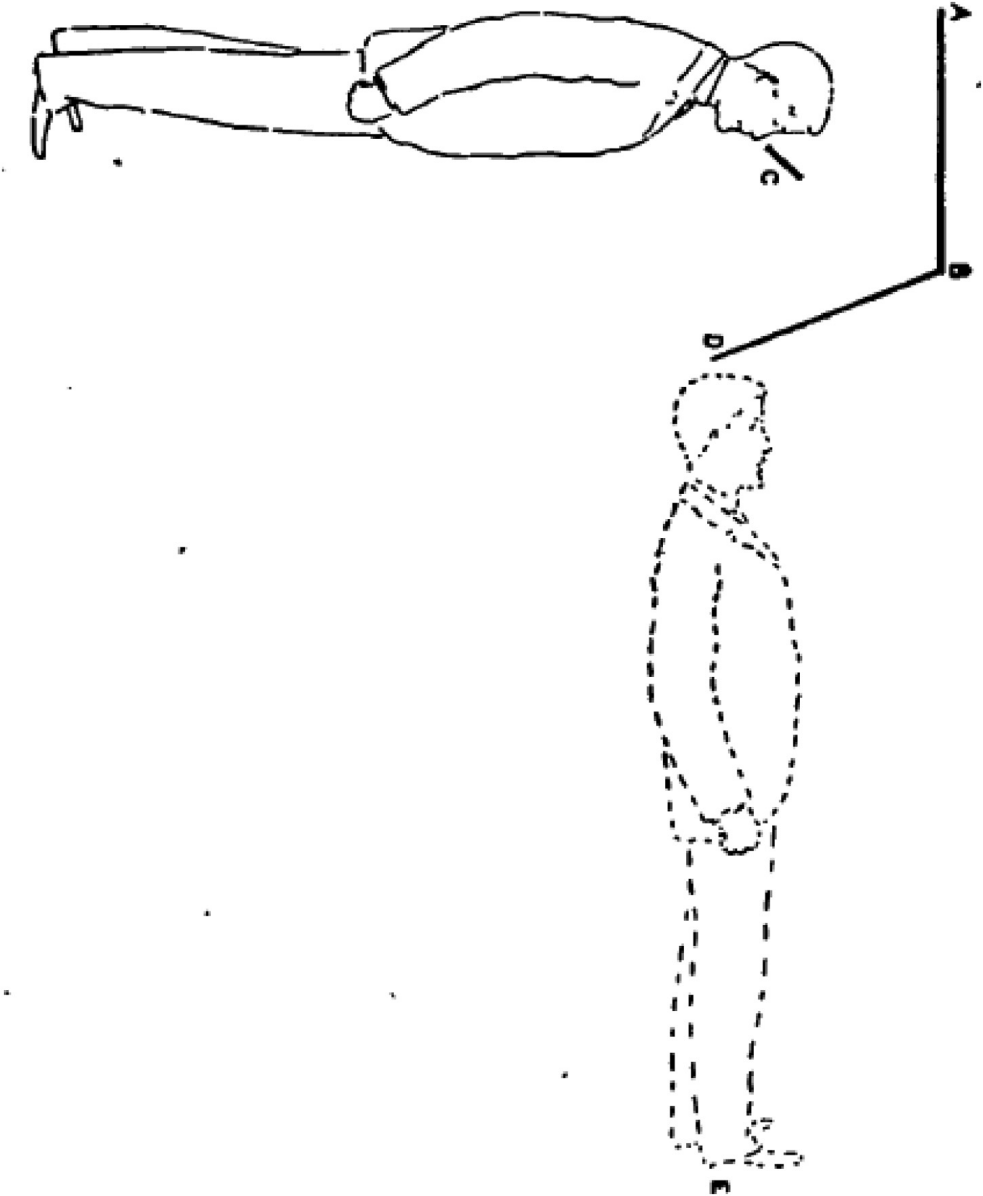
The three mirror set-up worn by Stratton (1899) gives the impression of seeing oneself from above as if floating horizontally in front of oneself.

**Figure 5.**
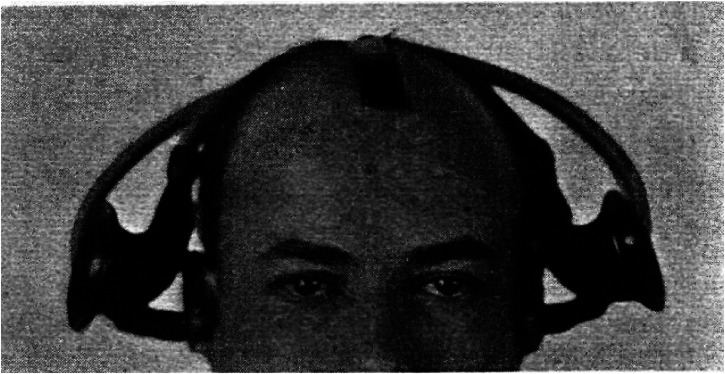
The distorting auditory pseudophone worn by Young (1928) to introduce sensory incongruency.

When discussing Höller's work, such as his well-known “Upside-Down Goggles” (1991–2001), the artist has been quoted as saying that “Subjective personal experience in science is a no-no” (in Douglas, 2011).^8^ It should, however, be stressed that the subjective report was, in fact, a key element of early perception research in which the effects of various distorting apparatus, such as inverting spectacles, were studied (e.g., Kohler, 1962; Stratton, 1897; Young, 1928). Stratton (1899) also explores experience with a configuration of three mirrors designed to give himself the sensation of seeing himself floating horizontally in front of himself while standing upright (see Figure 4). Meanwhile, Young's (1928) classic study involving the author wearing a pair of reversing pseudophones on his head (which effectively transposed the input to the two ears; see Figure 5) is also peppered with the author's first-person anecdotal subjective report documenting the process of adaptation and visual dominance (see also Bermejo et al., 2020; Willey et al., 1937).

There is, then, a certain irony, presumably lost on Holler himself that the installation used to illustrate the absence of the subjective in science (*Upside-Down Goggles*, 1991–2001) is actually based on a psychological investigation that was itself originally built entirely around the subjective report of the authors/scientists concerned. That being said, one can also turn things around, and note how the experimental research on which the artworks are loosely based, built as they are on the introspective reports of participants involved may also help to explain the pleasure resulting from the brain's attempt to integrate the sometimes-incongruent multisensory cues. Similarly, the sensory hallucinations that have often been documented in laboratory studies of sensory deprivation (e.g., Merabet et al., 2004; Motluck, 2007) also make an appearance in contemporary artworks such as Höller's “Psycho Tank” or Till Bödeker's “Think outside the Box” (Lohe, 2020) that have attempted to eliminate bodily sensation. It is perhaps also worth bearing in mind here how various principles of perceptual organization have been shown to feature in visual representational art (e.g., Conway & Livingstone, 2007; Van de Cruys & Wagemans, 2011; Wagemans, 2015), hence perhaps emphasizing the sometimes close connection between art and science.

Sensory incongruity is an important component of the success of a number of fairground rides, including the “haunted swing” illusion/ride, first described by Wood (1895). The latter experienced by Wood at the Midwinter Fair in San Francisco (and thereafter discussed in a brief article that appeared in a psychology journal). The deliberate introduction of sensory incongruity is also a distinctive feature of the laboratory perception research mentioned above (Young, 1928), as well of works such as “Upside-Down Goggles,” 1991–2001. Another of Höller's works, The Pinocchio Effect (1995) plays off the well-known Pinocchio illusion (e.g., Burrack & Brugger, 2005; Goodwin et al., 1972; Lackner, 1988; Lawton, 2007). Once again, though, just as in the case of “Upside-Down Goggles,” the work would appear to do little more than representing a scientific illusion that was originally discovered (and extensively studied) in the science laboratory and place it in an art gallery. It seems like something similar may be involved in “Rabbit on the Skin” (1996/2011) which tickles the visitor's forearm through the push of a button. And while the effort should not determine a work's status (consider here only the “readymades” of Marcel Duchamp; Goldsmith, 1983), I can't help but feel like these works would be equally well placed in a museum of illusion (e.g., https://www.museumofillusions.com/), or as part of public understanding of science-type “edutainment” (Podestà & Addis, 2007; Singhal & Rogers, 1999) exhibit. In fact, this is exactly where we presented a number of such illusions more than 20 years ago as part of The Royal Society's Annual Summer Science Exhibition in London (e.g., see Spence et al., 2001; see Szubielska et al., 2021, on the role of the physical context on the aesthetic experience of interactive installations).

**Figure 6.**
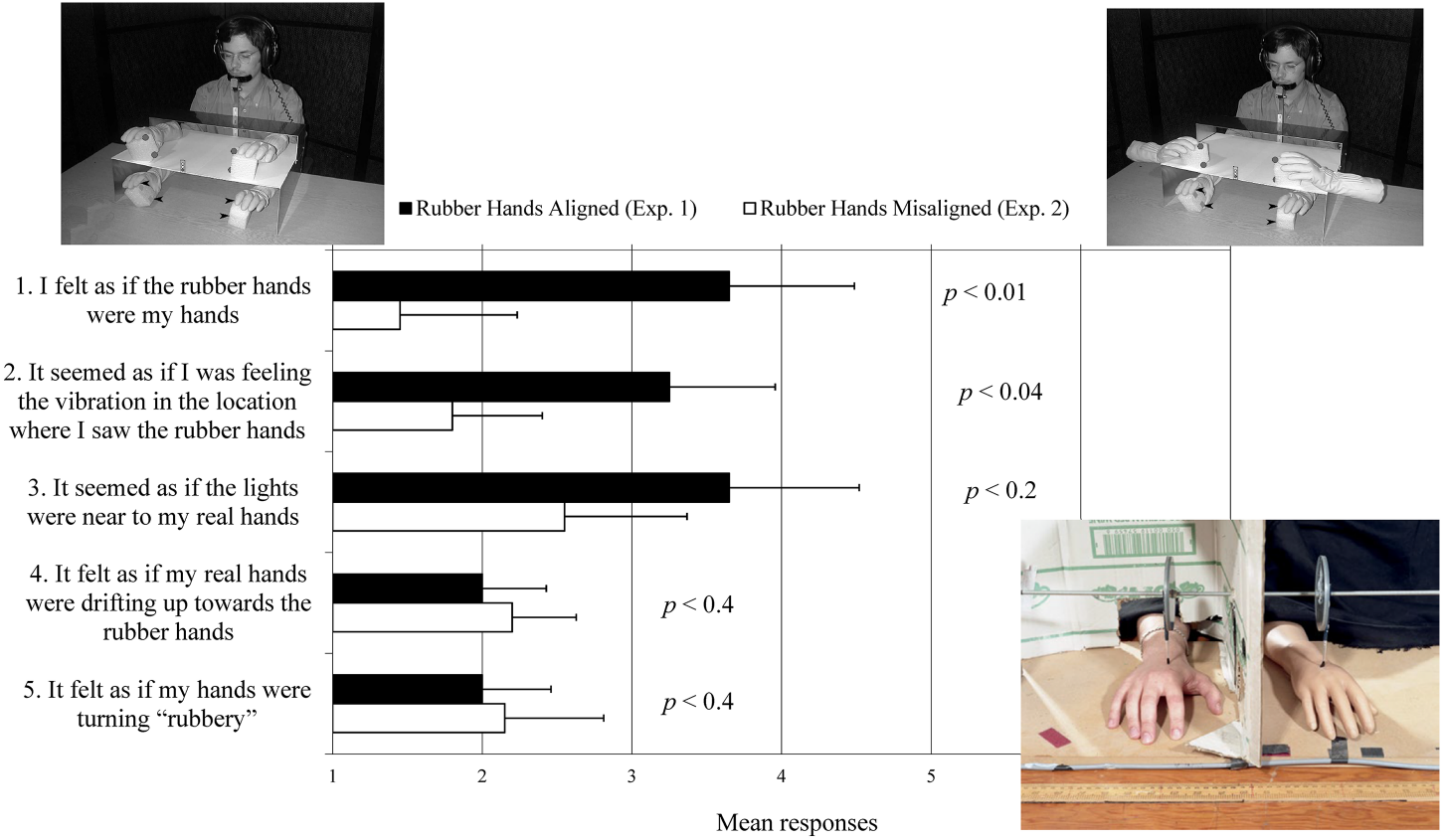
Photographs showing the experimental setup used in Pavani et al.'s (2000) study of out-of-body experiences while wearing rubber gloves. The photos highlight the location of the vibrotactile stimulators (indicated by arrows), visual distractor lights (dark circles on the upper cubes), when the rubber hands were present and aligned with the participant's own hands (Experiment 1, shown left), and when they were orthogonal to the participant's own hands (Experiment 2, shown right). The graph highlights the results of the subjective report questionnaire from the two experiments. (Error bars indicate standard errors of the mean.) Such results can be taken as arguing against Carsten Höller's suggestion that scientists are not interested in the subjective. Intriguingly, elements of the rubber hands research made their way into Daniel Stier's (2015), *Ways of Knowing*, photographic book documenting various scientific human experimental set-ups (shown bottom right). A number of the images in Stier's book share an intriguing similarity/confusability with several of the works appearing in Schwartzman’s (2011) *See yourself sensing: Redefining human perception*, hinting perhaps at the sometimes close proximity between artists and scientists working on the theme of (bodily) perception.

In other words, as one of the growing number of multisensory scientists who have spent a long time studying such bodily illusions (e.g., see Ehrsson et al., 2004; Michel et al., 2014, for a couple of examples), the artist's introduction of perceptual illusions into the gallery setting seems too easy. Importantly, much of the data collected from such experiments is very often of the subjective-report kind (i.e., “What does it feel like?”). Participants are asked to rate their agreement with a range of questions that probe their subjective experience (see Botvinich & Cohen, 1998). For instance, just take the questionnaire that the participants in one of our own studies (Pavani et al., 2000) were requested to complete more than two decades ago (see Figure 6). Once again, while objective performance data was also captured in this study, subjective report was a key part of the study's results. As such, Höller is incorrect to suggest that “subjective personal experience is a no-no” in science (see Douglas, 2011).

Books have been written on the theme of trusting the subject in psychological research (Jack & Roepstorff, 2008). And while unconstrained free report is often hard to analyze scientifically, directed questioning of a participant's subjective experience has proved a very fruitful source of information for many scientists. Perhaps, therefore, the key distinction here is not one between subjectivity and objectivity, but rather a question of who “learns from,” or is “affected by,” the experience. Notice how no data are collected in such art exhibits. It is all about the visitor's personal multisensory experience (Velasco & Obrist, 2020). By contrast, the participants in scientific research often provide a subjective report of their experience, but it is the experimenter who ultimately stands to learn/gain by scientifically analyzing or directing the kind of subjective reports that come back from their participants.

**Figure 7.**
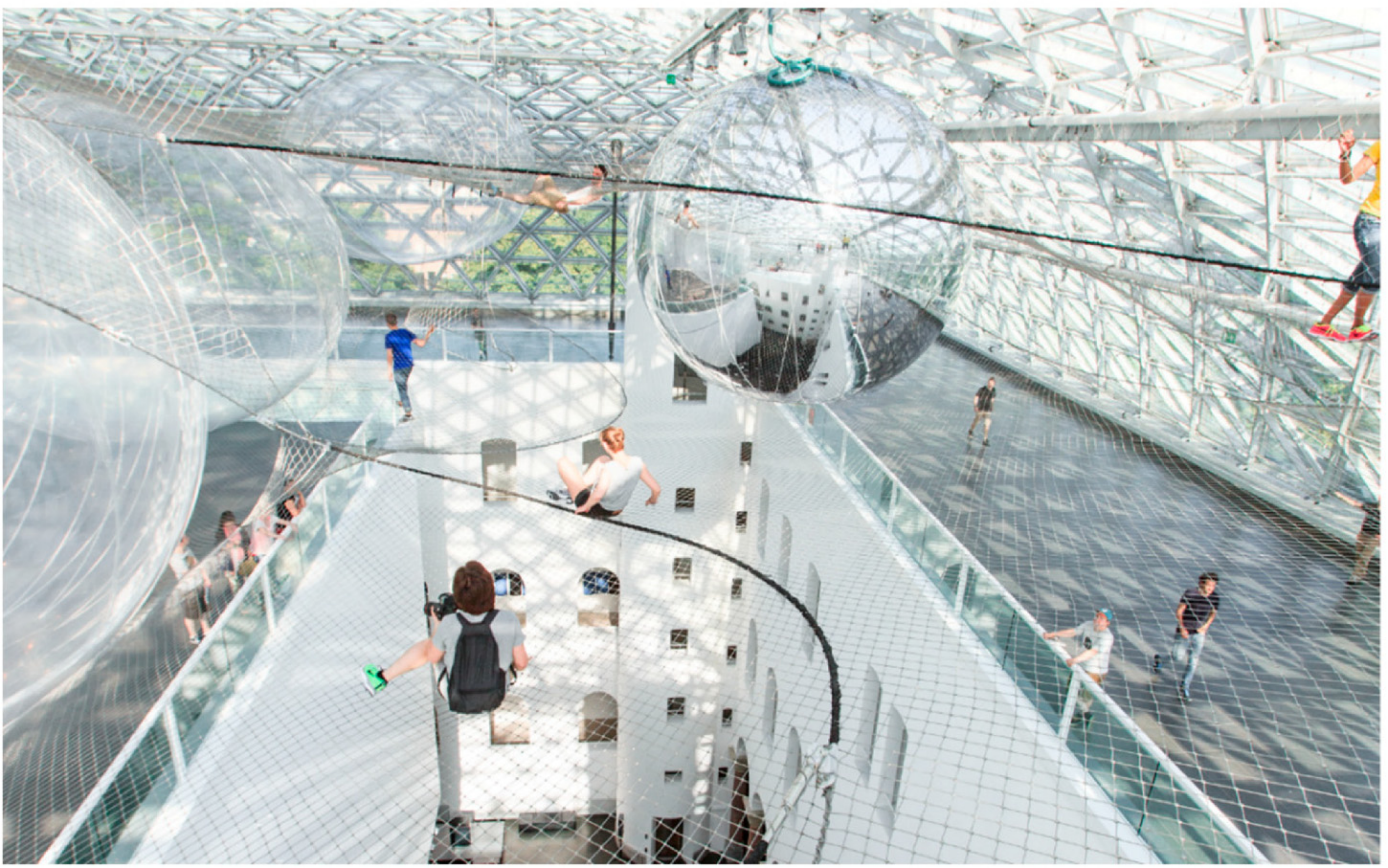
Tomás Saraceno, In Orbit, 2013. Solo exhibition at Kunstsammlung Nordrhein-Westfalen, K21 Ständehaus, Düsseldorf, Germany. Curated by Marion Ackermann and Susanne Meyer-Büser. Courtesy the artist; Tanya Bonakdar Gallery, New York; Andersen's Contemporary, Copenhagen; Ruth Benzacar Galería de Arte, Buenos Aires; Pinksummer contemporary art, Genoa; Esther Schipper, Berlin. © Photography by Studio Tomás Saraceno, 2013. [Reprinted from Wattolik (2019).].

Having myself experienced, Saraceno's “In Orbit” at K21 in Düsseldorf recently (Spence, 2022a), I must admit that I was rather more impressed both for the novelty and for the fact that the foregrounding of proprioceptive and vestibular sensations in this work appeared to be about more than simply itself (i.e., the phenomenology of the, possibly illusory, bodily senses). That is, one literally feels both the connection to others over the net (as it wobbles as others clamber over the work; Saraceno, 2015), while at the same time being suspended over the large fall, literally drawing one's attention to one's own mortality (playing with a version of the visual cliff, popularized by Gibson & Walk, 1960) (see Figure 7). That said, it is perhaps worth highlighting the fact that proprioception, and “prop. art” did not appear in any of the information that surrounds/is associated with, this exhibition. Intriguingly, proprioception is not mentioned in Saraceno’s (2015) piece about his oeuvre. Though, as Tomás Saraceno himself makes clear, his work is as much about social interconnectedness (Saraceno, 2015). Nevertheless, it can be argued that implicit in the concept is the notion of feeling connected through vibration, as in a spider's web, on which this work is modeled. As such, I would argue that “In Orbit” qualifies as an example of proprioceptive art, given that the work would lose some of its meaning were the proprioceptive/bodily feeling to be absent.

## Can Bodily Sensations be “Disinterested” in a Kantian Sense?

One challenge to considering at least certain works as prop art comes from pursuing the Kantian (Kant, 1892/1951) suggestion that aesthetic judgments are characterized by their disinterested nature (along with their subjectivity and their universality). Along similar lines, in their review of the scientific literature on visual aesthetics, Palmer et al. (2013, p. 81) argued that aesthetic judgments are: “‘disinterested’ in the sense that they do not involve desire. Preferring a larger to a smaller piece of cake would not count as an aesthetic judgment in Kant's framework, because such a judgment is (presumably) about one's desire to consume the larger one.” In what sense can the individual descending down through one of Höller's massive slide installations really be said to be disinterested? The artist himself describes “the experience of sliding is best summed up in a phrase by the French writer Roger Caillois as a ‘voluptuous panic upon an otherwise lucid mind.’ The slides are impressive sculptures in their own right, and you don't have to hurtle down them to appreciate this artwork. What interests Höller, though, is both the visual spectacle of watching people sliding and the ‘inner spectacle’ experienced by the sliders themselves, the state of simultaneous delight and anxiety that you enter as you descend.” (quoted on the Tate website, https://www.tate.org.uk/whats-on/tate-modern/unilever-series/unilever-series-carsten-holler-test-site).

In another interview, Höller notes that “People coming down the slides have a particular expression on their faces, they’re affected and to some degree ‘changed.’” It can be argued that such transformative experiences would not obviously seem to be disinterested.^9^ Here, it is perhaps worth returning to Höller's Mirror Carousel (2005), which, as some have noted, turns maddeningly slowly. On the one hand, this slowing down of the action helps to distinguish this example of bodily sensation from the white-knuckle excitement of many theme park rides (see Anderson & Burt, 2017; Spence, 2022c). The latter, presumably would not meet Kant's “disinterested” criterion, but the slow movement of the carousel in the museum might.

While I found myself clambering around on Saraceno's “In orbit,” exhibit, I must admit that I was similarly dubious of my ability to get beyond the fear of falling/instantaneous death (vertigo; cf. Thompson & Amedee, 2009), given the lack of obvious support the installation provides. Although one may know intellectually that the installation must be safe (and much of the text on the side of the exhibit was dedicated to stressing this fact), nevertheless, the thought of suddenly plummeting to the hard floor of the piazza more than 25 m below was never far from center stage in my thoughts. Perhaps if I had been allowed more than 10 min of so each group of up to seven people are allowed on the work then the answer might have been different. Or, as Lindblad (2012) suggests, many of these works really require a repeat visit to be appreciated properly. That said, there are other works that have people clambering through unfamiliar spaces using their hands to feel their way that do not trigger the vertigo-like response (e.g., trg. Transient Reality Generators, 2005. KIBLA Multimedia Center, Slovenia; see Schwartzman, 2011, p. 97). It remains a question for idle speculation as to whether such proprioceptive artworks are anything like as effective/successful as those that do trigger a very visceral vertiginous response.

At this point, it may be relevant to consider the long-running debate between Noël Carroll and Robert Stecker concerning the question of whether it is a necessary condition of aesthetic experience, in general, that it should be valued in its own right. Carroll denies the claim, Stecker, and more recently Durà-Vilà (2016) have argued in support. The latter point of view can be seen as standing in contrast to the Kantian notion of disinterestedness. As Durà-Vilà (2016, p. 95) notes: “Putting to one side Kantian disinterestedness, the idea that our aesthetic satisfaction should determine our aesthetic judgement is one that seems perfectly sound, current and popular.” He concludes that “beings such as ourselves cannot attend with understanding to objects suitable for aesthetic contemplation without experiencing them in such a way that we value the experience for its own sake.” (Durà-Vilà, 2016, pp. 98–99).

At this point, it may be worthwhile to try and distinguish between proprioceptive art and proprioceptive aesthetic experiences. In this article, I have put forward the suggestion that certain works might elicit interesting proprioceptive experiences without that necessarily being the intended focus of the artist (though, as we have just seen, the latter is undoubtedly, or can be, problematic). Here, it may be helpful to consider Jerrold Levinson's (1979, 1989, 2002) definition of art, since his approach captures both the relevance of the artist's intentions while, at the same time, also referencing how artworks are intended to be situated relative to previously established modes of appreciation. Returning to an earlier point, it is noticeable how a number of Holler's works are seemingly proprioceptive aesthetic experiences, which are based on the result of multisensory integration. In a number of such cases, the aesthetic experience would appear to reside primarily in the pure pleasure of the experience (e.g., of slow rotational motion on the merry-go-round, or the thrill of descend in Holler's large-scale slide installations).

## Eliminating the Body from the Experience of Proprioceptive Art

On the opposite extreme from those works that draw attention to the bodily senses, one might consider the various flotation tank installations, such as Carsten Höller's “Psycho Tank” or Till Bödeker's “Think Outside the Box” (see Table 1). In such cases, the artist effectively tries to remove, rather than to draw attention to, bodily sensations. The basic idea is to have the individual floating supine in a pool of water saturated with Epsom salt. The experience is typically calibrated so that sensory signals from visual, auditory, olfactory, gustatory, thermal, tactile, vestibular, gravitational, and proprioceptive channels are minimized, as is most movement and speech.^10^ Once again, though, if one looks at the long history of sensory deprivation research then it very soon becomes clear that the researchers working in this area have also long been interested in subjective reports of those who are sensorially deprived, be it in a flotation tank (e.g., Lilly, 1977; Zubek, 1969), or, more recently, simply by the extended use of blindfolding (e.g., see Merabet et al., 2004; Motluck, 2007).

Returning, though, to a point that was made a moment ago, contemporary research on many such sensory deprivation experiences explicitly assesses first-person report of the “What did it feel like?”-type. Once again, hinting at the fact that the subjective/objective divide is nothing like as clear in the psychological sciences as Höller (a biologist by training, remember) would have us believe. Taken together, then, the direct proprioceptive artworks would appear to either foreground bodily awareness (Shusterman, 2008), as in Saraceno's “In Orbit,” or else try to eliminate them altogether (as in the various flotation tank exhibits).

## Conclusions

There has undoubtedly been a rapid growth of interest in proprioceptive (prop) art in recent years, even if few of the artists themselves necessarily choose to use terms such as “proprioception” and “prop. art,” nor do they necessarily even directly refer to the stimulation of the bodily senses. There are likely a number of reasons for this growing interest in engaging the bodily senses: First, it is clear that the rise of the “experience economy” (e.g., Arrigo, 2016; Pine & Gilmore, 1998, 1999), and the increasing need for art galleries and other cultural institutions to justify their existence through encouraging increased footfall in their galleries makes entertaining the visitors that much more of an appealing proposition for the curator (e.g., Davis, 2015; Pursey & Lomas, 2018). In this regard at least, as Lindblad (2012) has noted, proprioceptive artworks, such as those of Carsten Höller undoubtedly succeed. However, the danger is that, at best, many such works simply end up foregrounding the peculiar bodily phenomenology associated with the multisensory effect or illusion itself (often resulting from sensory incongruency), rather than anything more meaningful or aesthetically resonant (see Lindblad, 2012). One can, in other words, think of this as a kind of commoditization of sensation (Mack, 2014).

On the other hand, the growing popularity of proprioceptive artworks/installations might also be contextualized in terms of the “shock of the modern” (Schivelbusch, 2014; cf. Bernard, 2014; Howard, 2021). Here, it might be fruitful to consider Sally Lynn's (2006) suggestion that the rise of proprioceptive pleasures and kinesthetic thrills (at the theme park), of a century or so ago can be seen as a direct response to the rise of disturbing new sensations that resulted from rapid technological innovation (such as the arrival of the railways; Schivelbusch, 2014; though see Starsmore, 1975, for a rather more mundane account). It is therefore open for one to wonder as to whether there might also be a contemporary shock perhaps linked to the rise of digital technologies capable of delivering a range of virtual and augmented sensations to the eye and ear (e.g., Eliot, 2021; Jütte, 2005; Velasco & Obrist, 2020).

Pursuing this idea still further, a number of the artistic works discussed in this review might perhaps be seen as a reengagement of, or reconnection to, the immediate bodily senses (Gallese, 2015).^11^ They might also be seen as fitting in within our growing awareness of the senses more generally (Merleau-Ponty, 1961; Paterson, 2021; Starobinski, 1989). Indeed, it is noticeable how limited is the stimulation of the bodily senses outside of the context of the exhibition space or theme park ride (see Parisi, 2018; Spence, 2022b). One might consider how such exhibits help to foreground the bodily senses that are so often neglected by those living in the online/digital world. There is also, perhaps, a blurring of the self-world distinction, and something about making people aware of the relationship between themselves and the world (Ratcliff, 2008; cf. Saraceno, 2015), that emerges from the best examples of proprioceptive art. Such foregrounding of the bodily senses might be seen (or should that be felt) to be especially important as we become increasingly disconnected by our addiction to the internet (e.g., Block, 2008; Flanagan & Booth, 2006)^12^ and increasingly to audiovisual life in the digital world or Metaverse (Parisi, 2018; Spence, 2022c).^13^

## Notes

1. To this list, a number of researchers would nowadays also want to include the pleasant touch system, involving the slow stimulation of the C-tactile afferents that are found predominantly in the hairy skin and which are tuned to the temperature of a human caress (e.g., see Ackerley et al., 2014).

2. For instance, Montero (2018) writes of the “embodied aesthetics” when dancers appreciate visually the performance of others. There are even those commentators out there agitating for rock-climbing to be considered as an aesthetic activity (e.g., Conroy, 2015; Nguyen, 2018), while others have chosen to focus on erotic works as a form of proprioceptive art instead (Benovsky, 2021).

3. The possibility that cross-sensory impressions of scent might be elicited in those viewing a painting of flowers also famously intrigued the French post-Impressionist painter Paul Cézanne (1839–1906; see Merleau-Ponty, 1964; Spence, 2020a).

4. This is especially apparent in the case of the so-called tactile art – artworks that are designed to be touched, and which galleries so often prevent from happening (i.e., as the artist intended; Bacci, 2011). For whatever reason, this does not seem to have happened in the case of those works that are prominently proprioceptive in nature. Perhaps, the intuition here is that the eye can reveal much of what the hand feels (though of course not all) thus leading to the (misguided) belief that not much is lost by preventing the visitor's touch, and so preserving the integrity of the artwork for future generations. By contrast, none of the other senses provide the same kind of information/experiences as the bodily senses.

5. He apparently gained a degree in biology, with a specialization in the communications systems of insects (Russeth, 2011).

6. Indeed, there has been something of a resurgence of interest in play. What is more, playfulness often seems to be linked to proprioceptive and kinaesthetic sensations (see Bridge, 2017; Grossmith & Grossmith, 1962, p. 103; Zimmerman, n.d.), including in an architectural context (see Pallasmaa, 1996, 2011; Spence, 2020b).

7. That said, there has been something of a turn away from relational aesthetics, a term popularized a quarter of a century ago now by Nicolas Bourriaud (Bourriaud, 1997), in recent years (see Russeth, 2011).

8. In an October 2011 interview with The Art Newspaper, Höller notes that “The real material I work with is people's experience […] I think of life as an experiment on oneself. Subjective personal experience in science is a no-no. In starting to make art, I wanted to bring in what had been forbidden.” (quoted in Douglas, 2011). As Lindblad (2012) notes: “Without recorded data or objective results, visitors are able to experiment with themselves freely, considering complex ideas and opening them up to the realm of possibility and personal discovery.”

9. One of the reviewer's of an earlier version of this article also suggested that those contemporary dance pieces that require audience participation, such as INVITED by the Belgian group Ultimate Vez, often involve lots of physical contact between dancers and the audience (see https://www.ultimavez.com/en/productions/invited). In such cases, it can also prove difficult to remain “disinterested” when engaging with such works.

10. Intriguingly, such tanks have, in recent years, taken on something of a role in the context of well-being treatment (e.g., Feinstein et al., 2018; Kjellgren & Westman, 2014; Suedfeld et al., 1983).

11. As Mandrou (1976) notes: “Until the eighteenth century at least, touch remained one of the master senses…It verified perception, giving solidity to the impressions provided by the other senses, which were not as reliable.”

12. At the same time, the idea that we might be disconnected from our tech. (as inevitably happens for those who enter the flotation tank) becomes an increasingly disturbing notion to many people nowadays (e.g., Moeller, 2012; Wilson et al., 2014).

13. As Smith (2008), the New York Times art critic noted of an exhibition of relational art held at the Guggenheim museum: “The larger point is to resensitize people to their everyday surroundings and, moreover, to one another in a time when so much—technology, stress, shopping—conspires against human connection.” Writing a few years earlier, Marks (2002, p. 149) notes: “The existential connection with the physical world is therefore quite attenuated in digital video.” Artists have responded in various ways to this disconnection from the senses (see also John Hudak “Short Work” https://turbulence.org/project/short-work/).
