# Addendum to “Proprioceptive art: How should it be defined, and why has it become so popular?”

**DOI:** 10.1177/20416695231191624

**Published:** 2023-08-07

Spence, C. (2022). Proprioceptive art: How should it be defined, and why has it become so popular? *i-Perception*, *13*(5). https://doi.org/10.1177/20416695221120522

The author wishes to acknowledge that some of the ideas around the concept of ‘proprioceptive art’ developed in the paper are also shared by Prof. M. Schrenk (https://proprioceptive.art/#:∼:text=(6)%20In%20proprioceptive%20art%2C,his%20or%20her%20exclusive%20audience) who was not explicitly mentioned by name in the article. While the acknowledgments to the paper thanked the organizers of the Prop Art workshop, which had proved very germane to the ideas developed in this chapter, Prof. Schrenk's work was not mentioned by name, for which the author wishes to apologize. Those readers wishing to find out more about Schrenk's thinking on the theme of proprioceptive art are directed to his 2014 book chapter (Schrenk, 2014).
